# Synthesis and Characterization of Chitosan/Reduced Graphene Oxide Hybrid Composites

**DOI:** 10.3390/ma12132077

**Published:** 2019-06-28

**Authors:** Karolina Kosowska, Patrycja Domalik-Pyzik, Małgorzata Krok-Borkowicz, Jan Chłopek

**Affiliations:** Department of Biomaterials and Composites, Faculty of Materials Science and Ceramics, AGH University of Science and Technology, Al. Mickiewicza 30, 30-059 Krakow, Poland

**Keywords:** graphene oxide, reduced graphene oxide, chitosan, lactic acid, acetic acid, tannic acid, hydroxyapatite

## Abstract

Graphene family materials (GFM) are currently considered to be one of the most interesting nanomaterials with a wide range of application. They can also be used as modifiers of polymer matrices to develop composite materials with favorable properties. In this study, hybrid nanocomposites based on chitosan (CS) and reduced graphene oxide (rGO) were fabricated for potential use in bone tissue engineering. CS/rGO hydrogels were prepared by simultaneous reduction and composite formation in acetic acid or lactic acid and crosslinked with a natural agent—tannic acid (TAc). A broad spectrum of research methods was applied in order to thoroughly characterize both the components and the composite systems, i.e., X-ray Photoelectron Spectroscopy, X-ray Diffractometry, Attenuated Total Reflection Fourier-Transform Infrared Spectroscopy, Scanning Electron Microscopy, ninhydrin assay, mechanical testing, in vitro degradation and bioactivity study, wettability, and, finally, cytocompatibility. The composites formed through the self-assembly of CS chains and exfoliated rGO sheets. Obtained results allowed also to conclude that the type of solvent used impacts the polymer structure and its ability to interact with rGO sheets and the mechanical properties of the composites. Both rGO and TAc acted as crosslinkers of the polymer chains. This study shows that the developed materials demonstrate the potential for use in bone tissue engineering. The next step should be their detailed biological examinations.

## 1. Introduction

A graphene, two-dimensional, one atom thick, sheet of carbon is a fascinating nanomaterial that has gained a lot of research attention recently, as graphene and its derivatives can be used in many different applications, including those for biomedical purposes [1,2,3]. This is also due to the fact that they can be chemically or structurally modified to alter or adjust their properties to specific needs [4,5]. Graphene oxide (GO), unlike graphene, has functional groups, e.g., carboxylic acid, epoxide, and hydroxyl groups, attached to a carbon sheet. Thermal, chemical or UV treatment of GO results in the formation of reduced graphene oxide (rGO) with a decreased number of oxygen groups and increased electrical conductivity [6]. rGO can be further exploited as a modifier of a polymer matrix to form nanocomposites with favorable properties [5,7,8,9,10]. Due to the unique properties, graphene and its derivatives have been widely tested in many applications, especially in biomedicine, as drug carrier materials and scaffolds [11,12]. Many research groups studied the toxicity of GO and rGO using different types of cells [13,14,15]. The mechanism of interaction of graphene derivatives with cells is still not fully understood but in the case of GO, its toxicity is determined by many factors, including the amount and type of oxygen-containing functional groups, as well as the size, charge, and agglomeration of the sheets [16]. It should be noted that in the case of rGO, an additional factor appears, i.e., the type of reducing agent used. The most popular reducing agent for GO is hydrazine [13], but due to its high toxicity, it cannot be used in biomedical applications. Furthermore, the studies showed that rGO fabricated using Na_2_S was more toxic than GO, although another research revealed opposite results [17]. For this reason, L-ascorbic acid [18] and natural-based GO reducing agents from plants [9,19,20] and mushrooms [21] have been proposed as an alternative. It is generally accepted that substances of natural origin perform better in biomaterial-related applications. 

Polymer matrices used to fabricate nanocomposites can have different origins, but in terms of biocompatibility, versatility, and similarity to native extracellular matrix (ECM) components, natural polymeric materials, such as polysaccharides, are the most attractive class [22]. Chitosan is a wonderful example of a linear, semicrystalline polyaminosaccharide. This biopolymer is a derivative of chitin, the main component of exoskeletons of crustaceans, fungi, and insects, and is characterized by the degree of deacetylation (DD), i.e., the ratio of its two main units, D-glucosamine and N-acetyl-D-glucosamine [23,24,25]. Both DD and the molar mass of chitosan, together with the presence of amino and hydroxyl groups, affect its physicochemical and biological properties. As a biomaterial, it is biodegradable and biocompatible, moreover, it exhibits analgesic, antibacterial, antifungal, hemostatic, and mucoadhesive properties [25,26]. However, being a natural polymer, chitosan lacks the appropriate mechanical strength for more demanding applications, such as bone tissue engineering. The fabrication of chitosan-based nanocomposites with carefully selected modifying phases can be a solution here. 

Bone tissue engineering requires the scaffolds to be not only biocompatible, biodegradable, and preferably bioactive, but also to withstand forces higher than in the case of other tissues. Graphene family materials (GFM) homogeneously dispersed in the polymer matrix to form composites can improve their mechanical strength and also promote cell proliferation and differentiation, accelerating the regeneration of a bone defect [27]. However, when designing such composite materials factors, such as the GFM concentration and arrangement in a polymer matrix, interactions between the matrix, modifying phases, and other components should be carefully considered. 

Previously [9], we have tested different green reducing agents for GO and shown that L-ascorbic acid is the most effective. In this study, hybrid nanocomposites based on chitosan and reduced graphene oxide were fabricated for potential use in bone tissue engineering. The CS/rGO hydrogels were formed either in acetic acid or lactic acid-based systems to evaluate the influence of the chosen solvent on the interaction between rGO sheets and the polymer chains, as well as the final properties of the hybrid materials crosslinked with a natural agent—tannic acid (TAc). 

## 2. Materials and Methods 

### 2.1. Materials

Chitosan (CS) with a deacetylation degree > 90% and high molecular weight (M = 60000–800000 g/mol) was purchased from Acros-Organics, Morris Plains, NJ, USA. Hydroxyapatite (HA) was purchased from Chema-Elektromet, Rzeszów, Poland. L-ascorbic acid (L-AA), concentrated acetic acid (AAc, ≥95,5%), lactic acid (LAc, 88%), tannic acid (TAc), 0.1M sodium hydroxide (NaOH), hydrochloric acid (HCl) solutions and reagents needed for the preparation of simulated body fluid (SBF), and phosphate-buffered saline (PBS) solutions were obtained from Avantor Performance Materials Poland S.A., Gliwice, Poland. All chemicals were of analytical grade. 

### 2.2. Graphene Oxide (GO) and Reduced Graphene Oxide (rGO)

Graphene oxide (GO) was received from the Institute of Electronic Materials Technology (ITME), Poland and synthesized as described in Reference [9]. For reduction, an aqueous suspension of GO (0.01 mg/mL) was obtained by ultrasonication for 2 h. Next, 300 mg of L-AA was added to 300 mL of the dispersion and sonicated for another hour. The reduction process of GO was carried out in alkaline conditions. The pH of the solution was adjusted to 10 (by addition of 0.1 M NaOH) to provide colloidal stability of GO sheets through electrostatic repulsion forces. Next, the suspension was heated up to 70 °C and kept for 2 h under vigorous stirring. The color of the mixture changed from brown to black with the reduction progressing. Obtained rGO suspension was centrifuged, washed a few times with distilled water to remove excess L-AA, then froze (−80 °C, 24 h), and freeze-dried (72 h, Labconco FreeZone). 

### 2.3. Fabrication of Chitosan/rGO Composites

The chitosan-based hydrogels were synthesized by simultaneous reduction of GO and self-assembling of the nanocomposites components. In the first step, GO dispersion was prepared by sonicating 37.5 mg of GO powder for 2 h in 12 mL of distilled water with NaOH addition (pH 9–11). Next, 0.375 g of L-AA was added to the GO dispersion (GO/L-AA ratio was the same as during the synthesis of rGO powder) and the solution was heated up to 70 °C with continuous stirring for 2 h to start the reduction process. After that, the dispersion was added to CS solution (2.5 g in 38 mL of 5% AAc or LAc) containing 0.15 g of HA. Next, 0.25 g of TAc was added and the whole system was left on a magnetic stirrer for 24h at 40 °C to allow the further reduction of rGO and self-assembling of hydrogel’s components. The mass ratio of CS to modifiers was 2.0 to 0.03, 2.0 to 0.2, and 2.0 to 0.12 for rGO, Tac, and HA, respectively. The composition of films was selected to obtain optimal physicochemical properties. The preparation of the solution containing more than 1.5% wt. of rGO was difficult due to the high viscosity. Finally, the mixture was cast onto Teflon dishes and left at room temperature. Series of composites were prepared by changing the type of solvent and mixture ratio. Nanocomposites without HA and/or TAc addition were also synthesized. 

### 2.4. Characterization

#### 2.4.1. X-ray Photoelectron Spectroscopy (XPS)

X-ray photoelectron spectrometer (Vacuum Systems Workshop Ltd., Crowborough, East Sussex, United Kingdom) with Mg anode (1253.6 eV Kα radiation; 200 W X-ray excitation source; 3 × 10^−8^ mbar vacuum; 15° electron takeoff angle) in the constant analyzer energy mode (22 eV pass energy) was used for characterization of GO and rGO. 

#### 2.4.2. X-ray Diffractometry (XRD)

X-ray diffraction measurement of nanofillers and chitosan-based composites was performed using X’Pert Pro diffractometer (Malvern Panalytical, Worcestershire, UK) with Cu Kα X-ray sources (λ = 1.5406 Å). D-spacing of GO and rGO was calculated from the Bragg equation:

*nλ = 2dsin(θ)* (1)

where *n* is a positive integer, *λ* is the wavelength of the indecent wave and *θ* is the diffraction angle.

#### 2.4.3. Attenuated Total Reflection Fourier-Transform Infrared Spectroscopy (ATR-FTIR)

The chemical compositions of materials were analyzed using attenuated total reflection spectroscopy. The spectra were taken in the range 4000–600 cm^−1^, at resolution 4 cm^−1^ using Bruker Tensor 27 (Bruker, Poznań, Poland) equipment with a diamond crystal. 

#### 2.4.4. Mechanical Properties

The tensile properties of composites were measured using a universal testing machine (Zwick 1435, ZwickRoell GmbH & Co. KG, Ulm, Germany) with a 5 kN load cell, at a constant speed of 1 mm/min, following ASTM D 882 standard. The reported results were an average of at least five independent measurements for each type of composite. 

#### 2.4.5. In Vitro Degradation

In vitro degradation of composites was carried out in PBS solution at 37 °C. Samples were immersed in 15 mL of PBS solution and stored in an incubator for 6 weeks. Once a week, pH of PBS solution was measured and the medium was renewed. The weight loss was quantified as the change in the dried sample weight over time:

(2) $$Weight\;loss=\;\frac{\left(W_{0}-W_{t}\right)}{W_{0}}100\%$$

where *W_0_* is the initial weight of the sample and *W_t_* is the weight after degradation time.

#### 2.4.6. Scanning Electron Microscopy (SEM)

The morphology of the prepared composites was characterized by scanning electron microscopy (Nova NanoSEM 200, FEI, Eindhoven, Netherlands) with an accelerating voltage of 10 and 18 kV. Chemical compositions of nanocomposites after incubation in SBF was studied by EDS (FEI, Eindhoven, Netherlands) measurements. 

#### 2.4.7. In Vitro Bioactivity Test

Preliminary bioactivity test was performed using the SBF solution prepared according to the improved protocol described by Bohner [28]. The samples were placed in containers and SBF was added to each of them with constant material surface area (mm^2^)/liquid volume (ml) ratio (10:1). Containers were placed in an incubator at 37 °C for 1 and 2 weeks. After incubation, samples were rinsed with distilled water and dried at 37 °C for subsequent analyses. 

#### 2.4.8. Wettability

The wettability of the composite films was assessed by water contact angle (WCA) measurement using goniometer (Drop Shape Analyzer, KRÜSS GmbH, Hamburg, Germany) at room temperature. The sessile drop of deionized water was deposited on the surface and contact angle was calculated. For each type of material, six drops were measured and the average value was reported. 

#### 2.4.9. Determination of the Number of Free Amino Groups

The number of free amino groups of chitosan-based composites was determined by ninhydrin assay [29] to evaluate the cross-linking effect of rGO, HA, and TAc. Briefly, 1.5 mg of freeze-dried sample was heated up with 1 mL of ninhydrin solution in a water bath for 1 h. After cooling down to room temperature, the obtained solution was diluted in 5 mL of isopropanol/water (1:1) mixture. The optical absorbance was recorded with a UV-vis spectrophotometer (CE2502, Cecil Instruments Ltd., Cambridge, United Kingdom) at 570 nm. Various glycine concentrations were used to obtain a calibration curve. The number of free amino groups in the tested samples was proportional to the optical absorbance of the obtained solution and was calculated as:

(3) $$Amount\;of\;free\;amino\;groups=100\%-\left(\left(\frac{\left(C_{p}-C_{c}\right)}{C_{p}}\right)100\%\right)$$

where: *C_p_*—concentration of free NH_2_ groups in pristine chitosan; *C_c_*—concentration of free NH_2_ groups in composites. 

#### 2.4.10. Cytocompatibility

The CS composite solutions were cast into a 48-well plate and dried overnight at 37 °C. Next, samples were incubated in 0.5M NaOH solution for 4 h. After removing NaOH, samples were washed with distilled water three times for 10 min and dried overnight at 37 °C. Samples were sterilized by incubating with 70% ethanol at room temperature for 30 min and subsequently dried for 1 h at 37 °C. Cytocompatibility of the as-prepared samples was evaluated in a direct contact with MG-63 cells (European Collection of Cell Cultures, Salisbury, UK) cultured in Eagle’s minimal essential medium (EMEM, PAN BIOTECH, Aidenbach, Germany) supplemented with 10% fetal bovine serum, 1% penicillin-streptomycin, and 0.1% sodium pyruvate (all of them from PAA Laboratories Gmbh, Pasching, Oberosterreich, Austria). Tissue culture polystyrene (TCPS) served as a control. The cells (1.5 × 10^4^ cells/cm^2^) were grown on the tested materials for 24 h, 3 days, and 7 days. Their metabolic activity was measured via Alamar Blue assay [30]. Three samples of each type were tested. Briefly, 0.5 mL of reagent (10 % (w/v) Resazurin solution in PBS, Sigma-Aldrich) was added and incubated with cells for 4 h at 37 °C. After 4 h 100 μL from each sample were transferred to a black 96-well plate and resazurin reduction was measured via fluorescence (kex.—530 nm, kem.—590 nm; FLUOstar Omega, BMG Labtech, Ortenberg, Germany). The results are shown as a mean ± standard deviation.

## 3. Results

### 3.1. Chemical Reduction of GO

**Figure 1:**
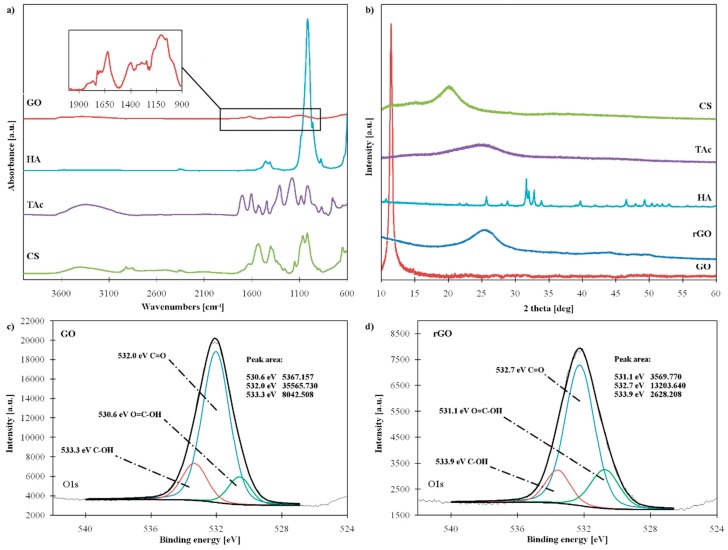
(**a**) ATR-FTIR (Attenuated Total Reflection Fourier-Transform Infrared) spectra, (**b**) XRD (X-ray Diffractometry) patterns of materials used for chitosan (CS) nanocomposites synthesis, and XPS (X-ray Photoelectron Spectroscopy) spectra of (**c**) graphene oxide (GO) and (**d**) reduced graphene oxide (rGO); (HA—hydroxyapatite, TAc—tannic acid).

All reagents used for the synthesis of nanocomposite hydrogels were characterized using ATR and XRD method (Figure 1a,b). Successful removal of oxygen-containing groups from GO was also confirmed using XPS (Figure 1c,d).

ATR spectrum of GO provides information about oxygen functionalities attached to the GO surface. The broad peak at 3387 cm^−1^ corresponded to -OH stretching vibrations. The peak of skeletal vibration from not oxidized C=C bond showed at 1620 cm^−1^. The characteristics peaks assigned to various types of oxygen-groups: C=O (stretching vibrations), C-O (stretching vibrations of C-O-C) and C-O (stretching vibrations of C-OH groups) can be found at 1723 cm^−1^, 1771 cm^−1^, and 1397 cm^−1^, respectively [18,31]. After the reduction process, the intensity of these groups was significantly reduced, which confirmed the successful removal of oxygen-containing groups from GO. 

Changes in the chemical compositions of GO were also examined by XPS. High-resolution O1s spectrum of GO (Figure 1c) showed three peaks assigned to different types of oxygen components: Oxygen of C-O (533.3 eV), oxygen of C=O (532.0 eV) and oxygen of O=C-OH (530.6 eV) [21,31]. In the O1s spectrum of rGO, the same peaks can be observed but their intensity is significantly smaller compared to GO, as shown in Figure 1d. The oxygen peak area of rGO decreased by 63%. 

The GO and rGO were also characterized by XRD. GO presented a sharp, single peak at 2θ value 11.51° corresponding to an interlayer d-spacing 0.77 nm. In contrast, in the rGO pattern the typical GO peak disappeared and a new wide peak appeared at 25.70 (d-spacing 0.35 nm), as presented in Figure 1b. After reduction, the interlayer distance between the sheets significantly decreased as a result of the removal of oxygen-containing functional groups [32,33]. 

### 3.2. Characterization of Nanocomposite Hydrogels

**Figure 2:**
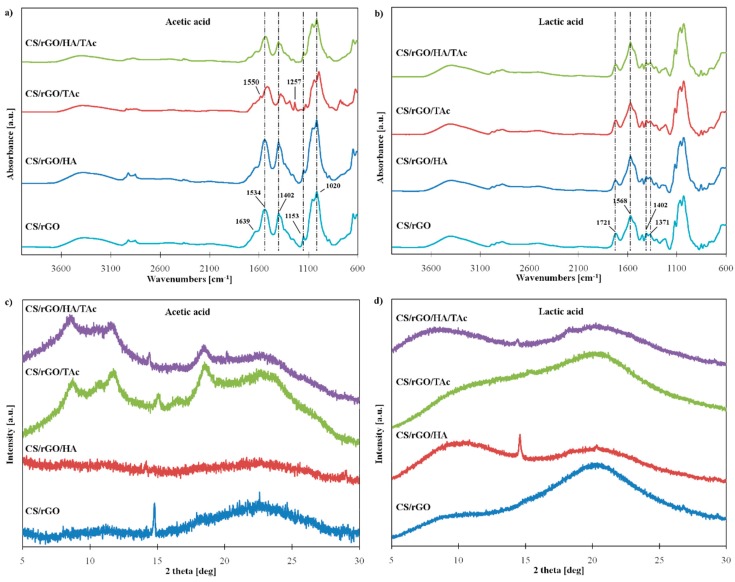
(**a**,**b**) ATR-FTIR (Attenuated Total Reflection Fourier-Transform Infrared) spectra and (**c,d**) XRD (X-ray Diffractometry) patterns of composite samples obtained from (**a**,**c**) acetic acid-based and (**b**,**d**) lactic acid-based solvent system (CS—chitosan, rGO—reduced graphene oxide, HA—hydroxyapatite, TAc—tannic acid).

To characterize components of the hydrogels, ATR and XRD measurements were done (Figure 1a,b). As previously demonstrated, GO contains oxygen-functional groups attached to the surface, which can potentially interact with chitosan groups. The characteristic peaks of CS in ATR spectra appeared at 1639 cm^−1^, 1534 cm^−1^, and 1402 cm^−1^ which corresponded to the C=O in the amide I (stretching vibrations in -NHCO-), N-H vibrations in NH_2_ group and CH_3_ deformations in amide groups, respectively. Peaks at 1153 cm^−1^ and 1020 cm^−1^ were attributed to asymmetric stretching in C-O-C bridge and stretching vibrations in the C-O group [34,35]. The characteristic peak of HA at 1015 cm^−1^ corresponded to P-O vibrations [36]. The spectrum of TAc reflects its complex structure. Peaks located at 1702 cm^−1^, 1443 cm^−1^, 1177 cm^−1^, and 753 cm^−1^ were attributed to the C=O, -C-C_aromatic_-, C-O stretching vibrations, and C=C distortion vibrations in benzene rings (32). The spectra of CS composites in AAc without TAc (CS/rGO and CS/rGO/HA) looked similar to the spectrum of pristine chitosan, as shown in Figure 2a. Characteristic peaks of GO and HA were not present, confirming the successful reduction of GO and good dispersion of the fillers in the matrix. The introduction of TAc significantly altered the spectra (CS/rGO/TAc and CS/rGO/HA/TAc). The smaller intensity of the peak corresponding to N-H band in -NH_2_ and shift from 1534 cm^−1^ to 1550 cm^−1^ can be related to the deformation of NH^3+^. Interaction of CS and TAc under acidic conditions caused the ionization of amine groups [37]. In CS/rGO/TAc spectra, new peaks showed up at 1639 cm^−1^, 1556 cm^−1^ and 1257 cm^−1^, which might be due to the interaction between components of composite and creating new amide (I, II and III) bonds. The ATR-FTIR spectra of the hydrogels prepared in lactic acid (Figure 2b) reveal some changes compared to the acetic acid systems. As seen in Figure 2b, the peaks corresponding to –CH_3_ deformation in amide groups at 1402 cm^−1^ and 1371 cm^−1^ decreased significantly. Moreover, the FTIR spectra of all the LAc samples show a large band at 1568 cm^−1^ attributed to -NH_2_. This shift of the amine group vibration to higher wavelength can be associated with the formation of a carboxylate between protonated -NH^3+^ of chitosan and –COO^−^ groups of LAc. The peak at 1721 cm^−1^ can be attributed to –COOH groups and suggest that free lactic acid is still present in the samples [38]. The FTIR results suggest that CS and LAc may interact with each other forming chitosan salt, i.e., chitosan lactate. 

The crystallinity of polymer is a very important factor, which determines its mechanical properties, as well as stability and biological response. CS can have three forms—amorphous, hydrated crystalline, and anhydrous crystalline. The pristine CS had a fully amorphous character, what can be identified by a broad peak centered at 20.07° (Figure 1b). The composite prepared by dissolving CS in AAc without TAc addition exhibited similar behavior, as shown in Figure 2c. The sharp peak at 14.81° in the CS/rGO pattern is the result of the anhydrous crystalline structure in the CS matrix. The composites with TAc (CS/rGO/TAc and CS/rGO/HA/TAc) showed a significant rise in the peaks assigned to crystalline forms of CS. Several peaks at ≈8, 11, 12° (hydrous forms) and ≈15, 18° (anhydrous forms) were found [39]. This phenomenon can be attributed to the synergistic effect of rGO and TAc as cross-linkers. rGO can support nucleation through exfoliation in alkaline conditions, what is connected with the increased surface area and strong electrostatic interaction with the polymer chains. All patterns of LAc-based composites exhibited an amorphous or almost fully amorphous character, even with TAc addition, as shown in Figure 2d. The single sharp peak at ≈15° corresponding to the anhydrous form of chitosan appeared in CS/rGO/HA (LAc) spectra. We speculate that the interaction between CS and LAc affected the packing of CS chains and resulted in amorphous forms of the composites. Peaks of rGO and HA did not appear in XRD patterns of composites, which confirmed good dispersion of fillers and strong interaction between CS and rGO. 

**Figure 3:**
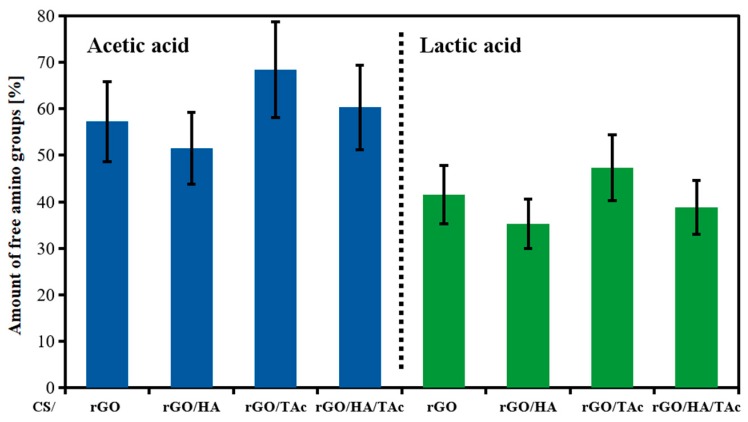
Amount of free amino groups in CS composites measured by ninhydrin assay (CS—chitosan, rGO—reduced graphene oxide, HA—hydroxyapatite, TAc—tannic acid).

Interaction between the components of the nanocomposites and amino functional groups of CS was also examined by ninhydrin assay (Figure 3). The addition of 1.5% (w/w) of rGO decreased the number of free amino groups in CS chains by more than 40%, confirming the reaction between functional groups attached to rGO surface and CS. Interestingly, the TAc addition reduced this effect. This allows to speculate that TAc interacted with rGO as well and impeded the cross-linking effect of rGO. A similar trend was observed for composite prepared in LAc, but the amount of free amino groups was smaller compared to AAc. This is consistent with ATR and XRD results suggesting an interaction between CS and LAc. 

**Figure 4:**
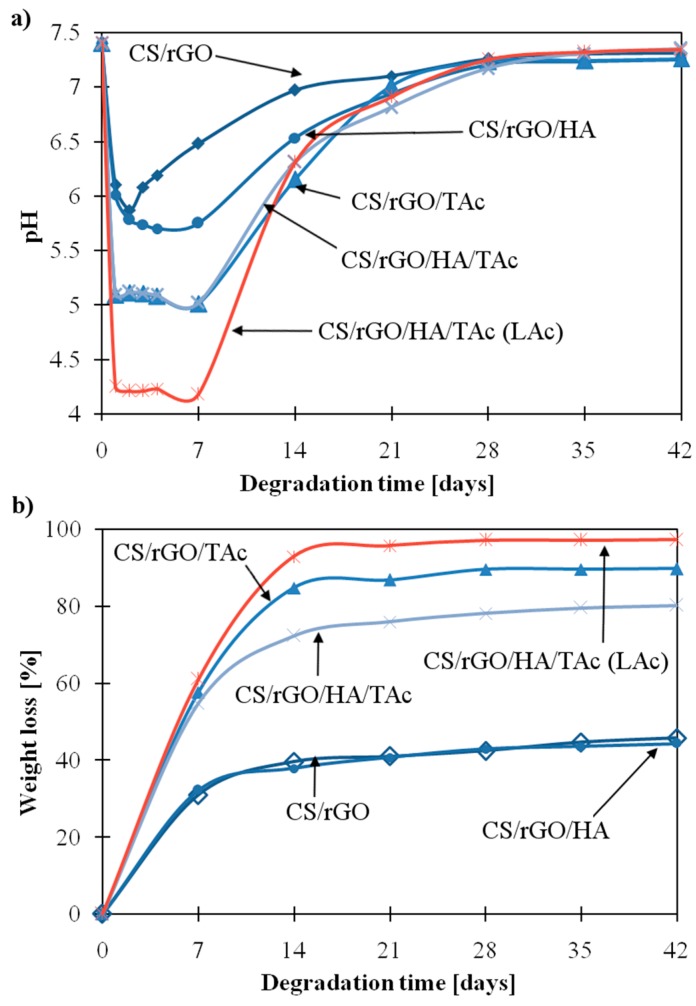
Degradation behavior of CS nanocomposites in PBS (Phosphate Buffered Saline) solution: (**a**) Changes of pH value of degradation media, (**b**) weight loss of samples during incubation, at 37 °C. Blue curves—composites prepared in AAc (acetic acid), red curve—composite prepared in LAc (lactic acid); (CS—chitosan, rGO—reduced graphene oxide, HA—hydroxyapatite, TAc—tannic acid).

The effect of rGO and TAc on the degradation rate of CS in PBS medium was also investigated. Pristine chitosan sample dissolved completely after one day. The composites prepared in LAc solution dissolved after one week, except for CS/rGO/HA/TAc. Figure 4a shows the change in pH of PBS solution during six weeks of degradation in 37 °C. In the case of CS/rGO (AAc), the pH value decrease was the least significant. After the first week, the pH decreased from 7.4 to 6.5. This is due to the removal of AAc from the sample. In the next step, the pH of medium returned to neutral value and the pattern plateaued. The degradation behavior of other samples prepared in AAc was similar but the decrease of pH value in the first week was more significant. In the case of CS/rGO/HA/TAc (LAc), change was more rapid and pH decreased to 4.1 in the first week. The degradation was also analyzed by measuring the weight loss of dried samples (Figure 4b). In the first step, the degradation rate was rapid for all composites and in the next, it was relatively smaller. The patterns plateaued after two weeks. The kinetics of degradation was the slowest for CS/rGO (AAc) and CS/rGO/HA (AAc). The weight loss after six weeks of degradation was ≈40%, while for CS/rGO/TAc (AAc) and CS/rGO/HA/TAc (AAc) it was ≈90% and ≈80%, respectively. The increased stability of CS/rGO (AAc) and CS/rGO/HA (AAc) can be explained by the presence of well-exfoliated rGO nanofillers in the polymer matrix. The mobility of the CS chains was limited by absorption onto the rGO surface through hydrogen bonding. In addition, rGO can act as a barrier for ions from the PBS solution. A faster degradation of the composites with TAc addition can result from limited interactions between CS and rGO, as concluded from the ninhydrin assay. The weight loss of CS/rGO/HA/TAc (LAc) was ≈95%. This can be related to the interaction between CS and LAc, which resulted in the formation of more soluble forms of CS. Other composites prepared in LAc dissolved completely after one week.

**Figure 5:**
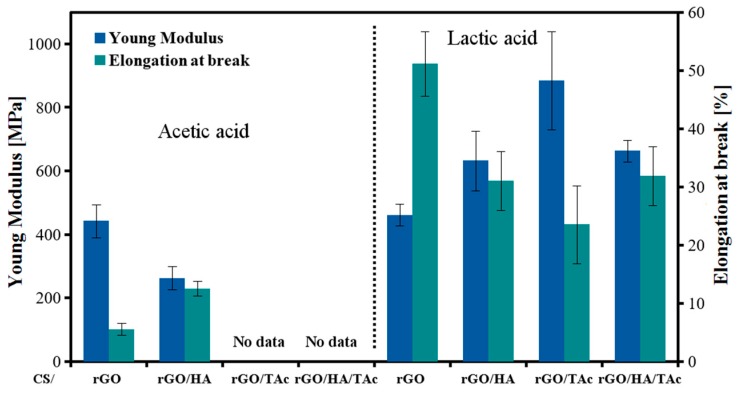
Young’s modulus and elongation at break of CS nanocomposites (CS—chitosan, rGO—reduced graphene oxide, HA—hydroxyapatite, TAc—tannic acid).

The mechanical behavior of the nanocomposite films was investigated by tensile test. CS/rGO (AAc) and CS/rGO/HA (AAc) were soft and easy to bend without cracking. The addition of TAc caused an increase in the degree of crystallinity and hence the fragility of the composites in a dry state. CS/rGO (AAc) and CS/rGO/HA (AAc) films showed balanced mechanical properties (Figure 5). The composites prepared in LAc solution behaved differently, what is believed to be related to their amorphous structure. The elongation at break of CS/rGO (LAc) exceeded 50%. At the same time, Young’s modulus of the film synthesized in LAc increased even by 50% compared to AAc-based composites. The positive effect of TAc was also noticeable. 

**Figure 6:**
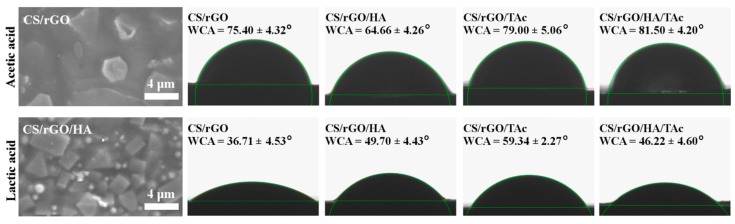
Representative SEM (Scanning Electron Microscopy) images of CS nanocomposites surface (CS/rGO prepared in AAc and CS/rGO/HA prepared in LAc solution) and representative images of water droplets on the samples’ surface with mean values of water contact angle—WCA (CS—chitosan, rGO—reduced graphene oxide, HA—hydroxyapatite, TAc—tannic acid, AAc—acetic acid, LAc—lactic acid).

Good mechanical properties of the composites are provided by specific, dense microstructure with rGO sheets arranged parallel to each other and the film’s surface (Figure 6) and strong π-π interaction between layers combined with strong interfacial adhesion between CS and rGO.

The surface wettability of nanocomposites is an important factor for tissue engineering. It was investigated with the water contact angle measurement. Due to the hydrophobic nature, the water contact angle (WCA) of pristine CS film was 109.71° ± 2.31°. Introduction of rGO into polymer matrix affected WCA significantly, as shown in Figure 6. The character of all samples prepared in the AAc solution changed to hydrophilic. The biggest improvement was observed for CS/rGO (AAc) (75.40° ± 4.32°). The rough film’s surface with arranged parallel—well visible under SEM—large rGO sheets was responsible for WCA improvement. This effect is even more significant in the case of the samples prepared in LAc. The WCA value of CS/rGO (LAc) was 36.71° ± 4.53°. 

**Figure 7:**
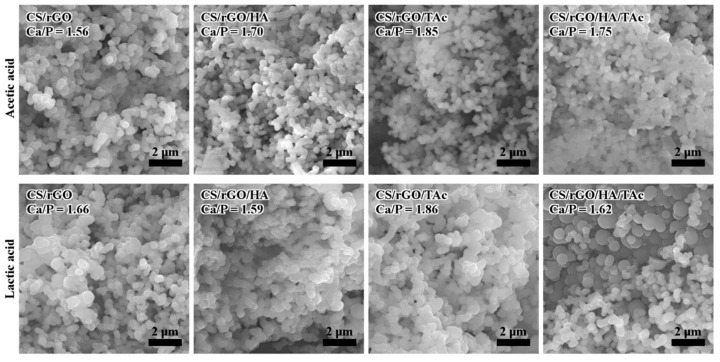
Representative SEM (Scanning Electron Microscopy) images of the surface of CS nanocomposites after 28 days of incubation in SBF solution (CS—chitosan, rGO—reduced graphene oxide, HA—hydroxyapatite, TAc—tannic acid).

**Figure 8:**
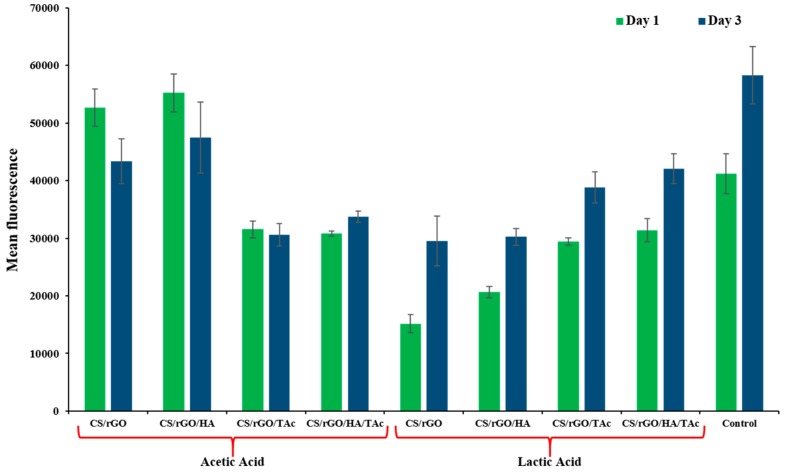
Alamar Blue assay—mean fluorescence after 24 h and 3 days (CS—chitosan, rGO—reduced graphene oxide, HA—hydroxyapatite, TAc—tannic acid).

The prepared nanocomposites were immersed in SBF solution to evaluate their in vitro bioactivity. The capacity to bond with living bone by the formation of an apatite interface layer on the material surface is an essential factor in tissue engineering. After 14 days of incubation, isolated groups of calcium phosphate particles were observed on all of the tested samples. SEM images in Figure 7 show that a dense, continuous layer of bone-like apatite was created on the surface of the nanocomposite after 28 days of incubation. Nucleated particles were spherical. Ca/P ratio was measured by EDS to investigate the chemical composition of the layers. The analysis confirmed that the Ca/P ratio of all of the layers was in the range 1.56–1.86 after 28 days, which is very close to the Ca/P ratio of HA. Interestingly, TAc addition resulted in an increase in the value of Ca/P ratio. Furthermore, the particles grown on nanocomposites synthesized in LAc had a slightly larger size. Preliminary in vitro tests confirmed cytocompatibility of the fabricated nanocomposites in contact with MG-63 cells (Figure 8).

## 4. Conclusions

The nanocomposites based on CS and exfoliated rGO were prepared successfully by a simple method with TAc as a crosslinker. Reduction of rGO during composite preparation allowed for the self-assembly of CS and rGO using π-π interaction between well-exfoliated rGO layers, which stacked in a special orientation, parallel to each other and the sample surface. This positively affected the wettability, mechanical properties, and stability of the composites. Crosslinking by TAc influenced the crystallinity of the composites, increased Young’s modulus and decreased the elongation at break. It also turned out that the type of solvent had a significant effect on the properties of the obtained hydrogels. A preliminary, in vitro biological test confirmed the cytocompatibility of the materials. More detailed examinations of the biological performance of the materials will be the next step.
